# *Listeria monocytogenes*—Can We Reduce or Eliminate It From Food Commodities?

**DOI:** 10.1002/mnfr.70329

**Published:** 2025-11-28

**Authors:** Loredana d'Ovidio, Débora Preceliano de Oliveira, Iskra Vitanova Ivanova, Manuela Vaz‐Velho, Bernadette Dora Gombossy de Melo Franco, Svetoslav Dimitrov Todorov

**Affiliations:** ^1^ ProBacLab, Laboratório de Microbiologia de Alimentos, Departamento de Alimentos e Nutrição Experimental, Faculdade de Ciências Farmacêuticas Universidade De São Paulo São Paulo Brazil; ^2^ Food Research Center Universidade De São Paulo São Paulo Brazil; ^3^ Department of General and Applied Microbiology Faculty of Biology Sofia University St. Kliment Ohridski Sofia Bulgaria; ^4^ CISAS ‐ Center for Research and Development in Agrifood Systems and Sustainability Instituto Politécnico de Viana do Castelo Viana do Castelo Portugal; ^5^ Department of General Hygiene I.M. Sechenov First Moscow State Medical University Moscow Russia

**Keywords:** antimicrobial, bacteriocins, environmental stress, *Listeria monocytogenes*

## Abstract

*Listeria monocytogenes* is a highly virulent foodborne pathogen responsible for listeriosis, a severe infection threatening vulnerable populations such as pregnant women, newborns, the elderly, and immunocompromised individuals. Its resilience, surviving low pH, reduced water activity, and refrigeration, makes it a formidable contaminant in food systems. Traditional control methods include heat treatment, high‐pressure processing, irradiation, and acidification, all aimed at reducing bacterial load in ready‐to‐eat foods. WHO and FAO guidelines emphasize minimizing contamination and growth. Emerging strategies target gene expression to curb virulence and survival. External signals like chitin can suppress pathogenic genes, while nucleomodulins alter host chromatin to disrupt infection. Regulatory proteins such as MogR and GmaR modulate motility‐related genes, and selective pressures from antimicrobials or bacteriophages can reshape bacterial behavior. Genetic tools like CRISPR‐Cas9 offer precision editing of key genes. Additional interventions include environmental adjustments (temperature, pH, salinity), bacteriophage applications (e.g., PhageGuard Listex, ListShield), and competitive exclusion via beneficial microbes. Natural antimicrobials like bacteriocins (nisin, pediocin, enterocin, plantaricins, and lactocin S) disrupt cell walls and membranes. Phenolic compounds such as allicin, eugenol, and curcumin also exhibit inhibitory effects. Combining these approaches is vital for effective control and enhanced food safety.

## Graphical abstract

*Listeria monocytogenes* is a foodborne pathogen responsible for listeriosis. Traditional control methods include heat treatment, high‐pressure processing, irradiation, and acidification, all aimed at reducing bacterial load in foods. Emerging strategies include gene expression, CRISPR‐Cas9, bacteriophage, competitive exclusion via beneficial microbes, natural antimicrobials like bacteriocins, phenolic compounds or combining these approaches.

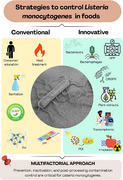

## 1 The Importance of *Listeria monocytogenes* for Food Safety and Public Health

Bacteria‐associated diseases are among the most prevalent food‐related clinical cases, affecting large portion of the global population [1]. Among these bacteria, *Listeria monocytogenes* is a foodborne pathogen that causes a serious infection (listeriosis) that primarily affects pregnant women, newborns, older adults, and individuals with weakened immune systems [2, 3]. *L. monocytogenes* is a non‐spore forming and rod‐shaped Gram‐positive bacterium recognized as one of the most virulent foodborne pathogens. Notably, it can grow at low temperatures, including under commercial refrigeration, and capable of surviving freezing storage [4, 5].

*L. monocytogenes* belongs to the family *Listeriaceae*, which comprises two genera: *Listeria* and *Brochothrix*. The genus *Listeria* has been divided into *sensu stricto* and *sensu lato* groups. Listeria *sensu stricto* group is composed of *L. monocytogenes*, *L. seeligeri*, *L. welshimeri*, *L. innocua*, *L. ivanovii*, *L. marthii*, *L. farberi*, *L. immobilis*, *L. cossartiae*, and *L. swaminathanii*. Within the sensu stricto group, only *L. monocytogenes* is considered pathogenic to humans and animals, and *L. ivanovii* is pathogenic only to ruminants. Currently, the Listeria *sensu lato* group has been used to refer to those Listeria species that are less phylogenetically and phenotypically related to L. monocytogenes, which are: *L. grayi*, *L. fleischmannii*, *L. floridensis*, *L. aquatica*, *L. valentina*, *L. thailandensis*, *L. goaensis*, *L. ilorinensis*, *L. costaricensis*, *L. rustica*, *L. portnoyi*, *L. cornellensis*, *L. newyorkensis*, *L. rocourtiae*, *L. weihenstephanensis*, *L. grandensis*, *L. booriae*, and *L. riparia*. Due to their phenotypic characteristics, some species within the *sensu lato* group are not considered reliable indicators of *L. monocytogenes* presence, as they are unable to grow under certain conditions where *L. monocytogenes* can. Therefore, it is recommended that only Listeria *sensu stricto* species to be used as indicators of a higher risk for *L. monocytogenes* presence in food and/or environment [6].

Whole genome single nucleotide polymorphism (SNP) analyses have shown that *L. monocytogenes* isolates comprise four divergent evolutionary lineages (I–IV) and can be further subdivided into sublineages (SL), 14 serotypes (i.e., 1/2a, 1/2b, 1/2c, 3a, 3b, 3c, 4a, 4 ab, 4b, 4c, 4d, 4e, 4 h, and 7) and sequence types (ST) which are then grouped into clonal complexes (CCs) and classified using core‐genome multi‐locus sequence typing (cgMLST or CT). While all *L. monocytogenes* strains are potentially pathogenic, epidemiological, and experimental evidence indicates that only three serotypes (1/2a ‐ lineage II; 1/2b and 4b ‐ lineage I) account for 92%–95% of human clinical isolates, although some serotype 4b strains belong to lineage III or IV. Lineage II isolates are mostly associated with food, whereas lineage I isolates are predominantly linked to clinical cases in Western countries. Interestingly, in China and Taiwan, serotype 1/2b is the most prevalent both in food and clinical cases, indicating that local factors contribute to the geographic diversity of *L. monocytogenes*. This underscores the importance of epidemiological surveillance in controlling and preventing the pathogen's occurrence in food [6, 7, 8, 9].

**FIGURE 1:**
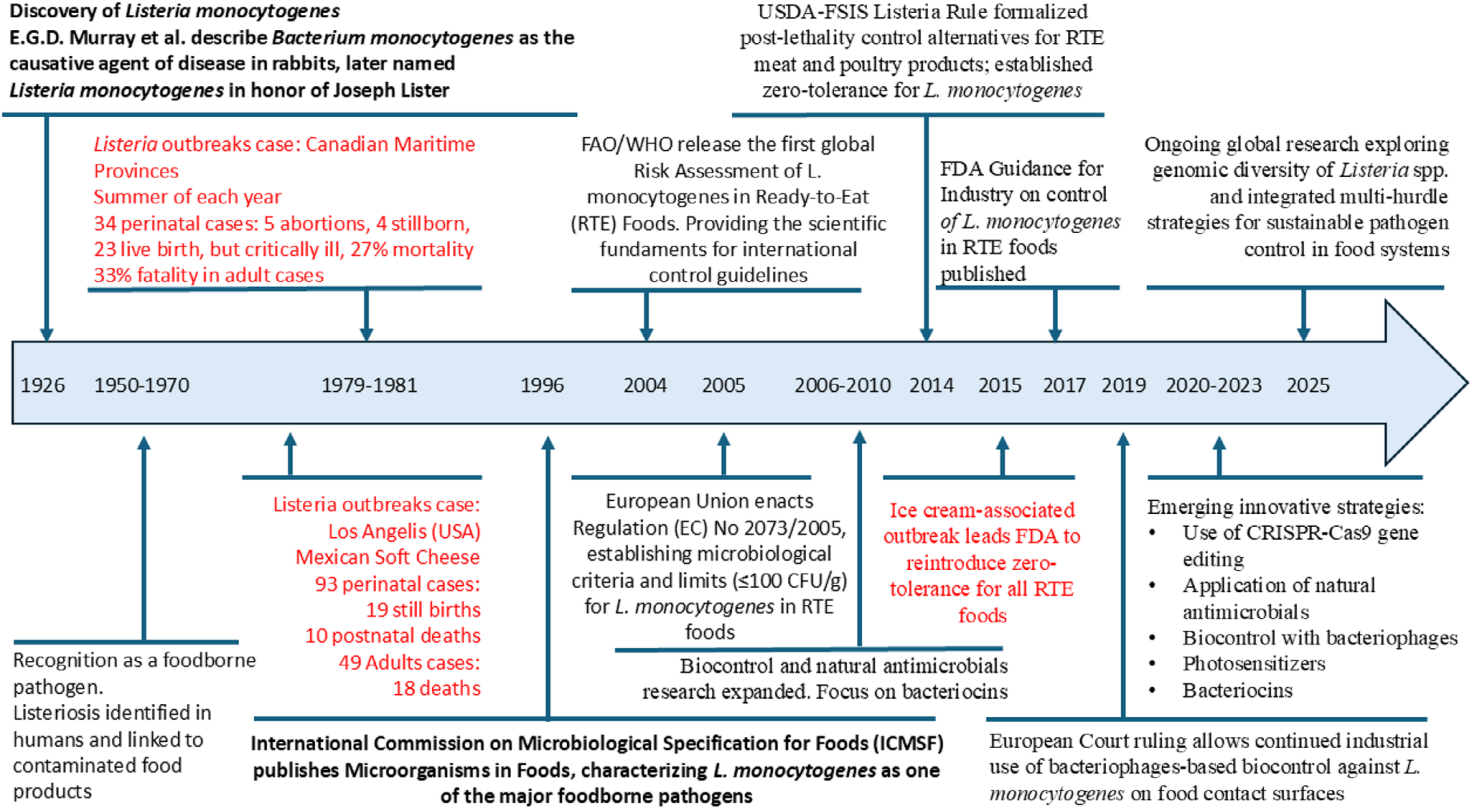
Timeline of principle key points in the history of *Listeria monocytogenes*, from discovery in 1926, via outbreaks and legislation to innovative approaches in control.

The first reports on *L. monocytogenes* date back to a century ago, to 1924, when E.G.D. Murray, a bacteriologist from Cambridge, isolated Gram‐positive rods from the blood of laboratory rabbits that had died suddenly. The new identified bacterium was initially named *Bacterium monocytogenes* [10, 11]. Later, in 1940, Harvey Pirie proposed renaming the genus to *Listeria* in honor of Joseph Lister [10] (Figure 1). Since then, numerous outbreaks and sporadic cases of listeriosis have been reported worldwide, linked to various food products such as cheese, meat, seafood, vegetables, and fruits. Even with all extensive research focusing on pathogenicity of *L. monocytogenes*, more research is still needed to better understand the epidemiology, pathogenesis, diagnosis, treatment, and prevention of listeriosis (Figure 1).

The unique physiology of *L. monocytogenes* makes it highly adaptable and a resilient foodborne pathogen. This bacterium is ubiquitous and can grow in a wide temperature range (−0.4°C to 45°C, with optimum of 37°C), including very low (refrigerated foods) [12]. It is resistant to a wide range of pH levels (4.5–9.5) as well as to tolerate salt concentrations up to 20% and is relatively unaffected by reduction of water activity (*a_w_* < 0.90) compared to other foodborne pathogens. Additionally, *L. monocytogenes* has the ability to form biofilms and can move intracellularly in host cells by utilizing actin filaments [2, 3, 4, 5, 12].

Some of the symptoms of listeriosis include fever, stiff neck, headache, vomiting, weakness, diarrhea, and confusion [13]. Listeriosis can be diagnosed through blood or cerebrospinal fluid cultures [9], and is typically treated with antibiotics such as ampicillin, gentamicin, or benzylpenicillin [14].

To prevent listeriosis, and most of the other food associated diseases, it is important to understand its features and epidemiology and practice proper food storage, handling, cooking and cleaning and sanitation practices [4]. However, the unique physiology of *L. monocytogenes* and its ability to grow and survive under adverse conditions make this microbial species a serious threat to public health and for the food industry [2, 3]. Foods that are at higher risk for listeria contamination include RTE meat products and others that usually are not cooked before consumption, like unpasteurized milk, soft cheeses, deli meats, raw and smoked seafood, and raw sprouts [4].

As a ubiquitous pathogen, *L. monocytogenes* is widely disseminated in agricultural, aquacultural, and food processing environments, where it readily contaminates food systems and/or food production environments [15]. At farm level, potential sources of the pathogen may include sewage contaminated‐water sources, irrigation water used for food crops, livestock drinking water, soil, pasture, bedding materials, animal feces, manure from asymptomatic animals used as fertilizer for produce, improperly fermented silage used for animal feed, udder surface of dairy ruminants, as well as farm workers, utensils and equipment [15, 16, 17, 18, 19].

Birds and wild animals are also considered additional sources for *L. monocytogenes* contamination on farms [19, 20]. When production animals develop a sub‐clinical gastrointestinal infection, the herd level prevalence of *L. monocytogenes* can exceed 90% and often accompanied by highly variable fecal shedding [21]. High stocking density further contributes to pathogen environmental dissemination and increases risk of infection among animals [17, 19, 22].

At slaughterhouses, these animals may carry *L. monocytogenes* on skin surface and/or in intestinal tract increasing the likelihood of contamination during meat processing and potentially introducing the pathogen into the broader food processing environment [22].

In food processing facilities, besides the contaminated raw material (meat, milk, or produce) as a potential source, other contamination sources for the final product are also present, especially because of the biofilm production capacity of *L. monocytogenes*, causing the pathogen persistence in the food environment, equipment and/or food contact surfaces. Inefficient cleaning and sanitation procedures, along with poorly hygienic designed equipment and/or facility layout that hinder effective cleaning and sanitation processes lead to accumulation of organic matter. This in turn creates a favorable condition for harboring, developing and maintaining *L. monocytogenes* populations ultimately increasing the risk of cross‐contamination [12, 15]. Similarly to food processing environments, retail and food services can also be a contamination source. Once the pathogen is in the environment and finds conditions that support its persistence, RTE cross contamination can occur [23].

The pathogen's physiological adaptability—including its ability to survive and growth at low temperatures, under low *a_w_*, across a wide pH range, and its tolerance or resistance to commonly biocides used in food industry also contributes to its survival [2, 3, 4, 5, 24]. This is particularly concerning the context of post‐lethality treatment contamination of RTE products, where no further step is available to inactivate the pathogen, thereby increasing the consumer exposure and risk of infection [15, 25, 26, 27].

## 2 Guidelines to Minimize and/or Prevent the Contamination and/or Growth of *L. monocytogenes* in Foods

**FIGURE 2:**
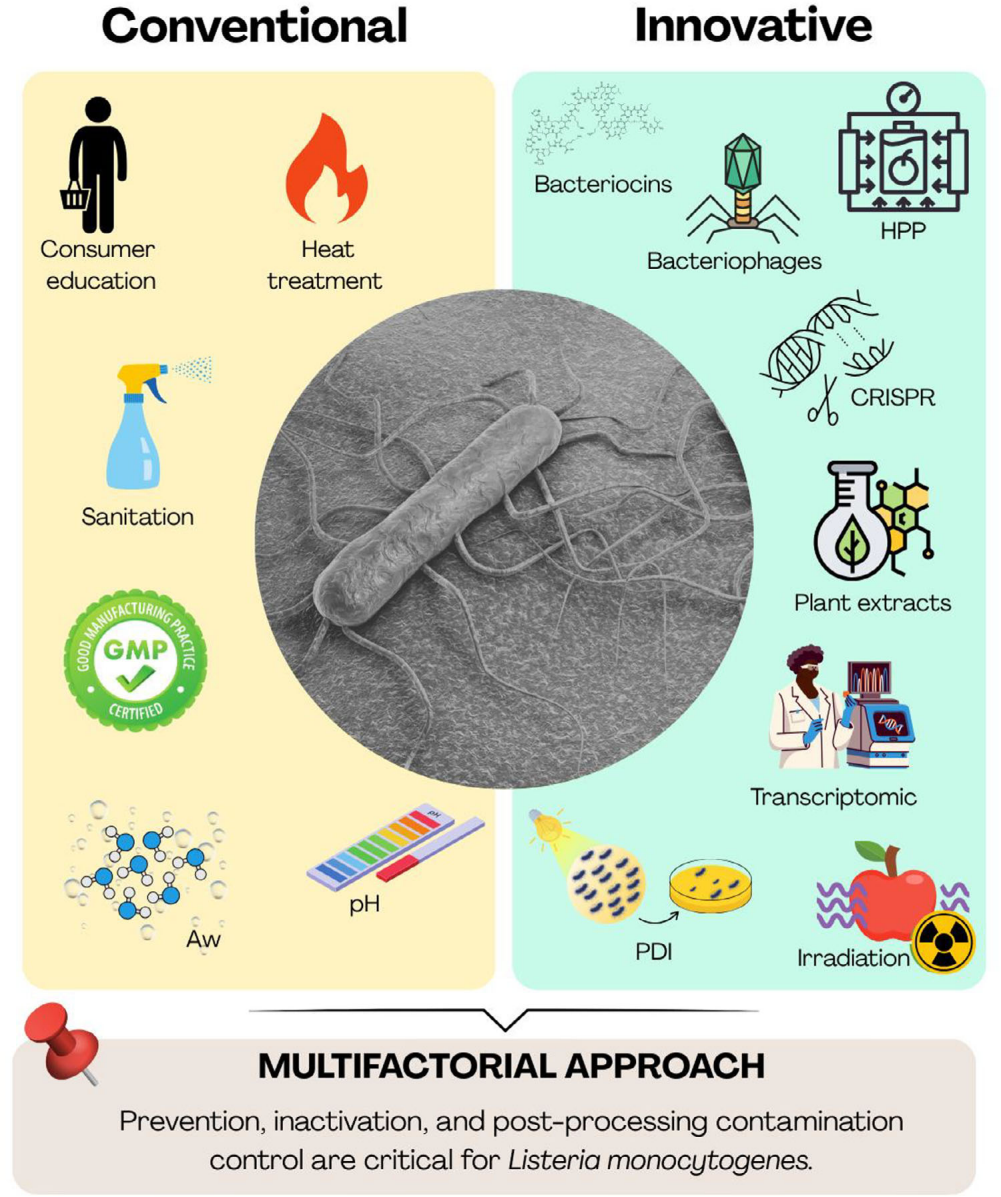
Strategies to control *Listeria monocytogenes* in food commodities.

Since the discovery of *L. monocytogenes*, its association with foodborne infections has driven researchers and the food industry to launch a concerted effort against this pathogen [2, 3, 4]. A key strategy is to prevent the presence of *L. monocytogenes* in food products by disrupting the connection between its natural reservoirs and the food supply [28]. The food industry employs several approaches to reduce or eliminate *L. monocytogenes* during food production (Figure 2). Moreover, strict hygiene practices and heightened awareness are essential to prevent external and cross contamination, especially during post‐lethality processing. This includes proper food storage, handling, and cooking, as well as routine cleaning and sanitization of the food processing environment. Implementation of high hygiene practices can help minimize cross‐contamination from raw materials, equipment, or personnel [29, 30].

As part of the food preparation and handling processes, appropriate listericidal steps can be applied in food processing and storage, to a safe threshold. However, a critical question remains: what constitutes a safe level of *L. monocytogenes* in RTE food products? Some regulatory agencies have developed guidelines based on the food product category, the ability of the food product to support pathogen growth during shelf life, heating prior consumption, infective dose, epidemiology data, and risk assessment analysis.

The World Health Organization (WHO) and the Food and Agriculture Organization (FAO) have conducted an international quantitative risk assessment of *L. monocytogenes* in ready‐to‐eat foods, providing scientific basis for the *Codex Alimentarius* Commission (CAC) to develop a document entitled “Guidelines on the Application of General Principles of Food Hygiene to the Control of *Listeria monocytogenes* in Foods.” The guidelines provide advice for governments worldwide for the control of *L. monocytogenes* in ready‐to‐eat food, aiming to protect public health and ensure fair practices in food trade [31, 32].

The guidelines are applicable to ready‐to‐eat foods and cover all stages from primary production to consumption. It is focused on control measures to minimize and/or prevent the contamination and/or growth of *L. monocytogenes* in this category of food products, considering the results of the mentioned WHO/FAO risk assessment of *L. monocytogenes* in ready‐to‐eat food products [31].

The risk analysis considered two scenarios of contamination limit (0.04 and 100 CFU/g) and concluded that the vast majority of listeriosis cases would result from the consumption of food with numbers of the pathogen where the contamination exceeded either limit considered for the risk analysis. In other words, when the compliance is 100% to the limit established, the predicted cases number is low for both limits, with a 10‐fold difference between them. The elimination of products with counts of *L. monocytogenes* above the limit established at the time of consumption was found to reduce the number of predicted illnesses cases [31].

The CAC guidelines also categorize ready‐to‐eat foods into two groups: ready‐to‐eat foods where growth of *L. monocytogenes* can occur, and those where growth will not occur (Table 1). For the category where growth can occur, the microbiological criterion is absence in 25 g (<0.04 CFU/g). These foods are defined as ready‐to‐eat items with a potential for a greater average increase of 0.5 log CFU/g of L. monocytogenes during the expected shelf life, distribution and storage. For the category of ready‐to‐eat foods where growth will not occur, the microbiological criteria is set at 100 CFU/g [32].

**TABLE 1:** Growth limits for *Listeria monocytogenes* [33].

	Minimum	Optimal	Maximum
Temperature	−0.4°C (31.3°F)	37°C (98.6°F)	45°C (113°F)
pH	4.39	7.0	9.4
Water activity (*a_w_*)	0.92	—	—

This limit is determined based on scientific justification and validated studies, considering product's intrinsic and extrinsic factors, such as pH, water activity (*a_w_*), freezing, added inhibitors, and so forth, either alone or in combination. Although *L. monocytogenes* will not grow under certain conditions, but it may still be able to survive.

In United States, the US Food and Drug Administration (FDA) and the US Department of Agriculture's (USDA) Food Safety Inspection Service (FSIS) have different approaches to food safety assurance regarding *L. monocytogenes*. In May 2003, FSIS issued the “FSIS Risk Assessment for *L. monocytogenes* in Deli Meats.” This risk assessment indicated that the use of a combination of growth inhibitors and post‐lethality interventions to control *L. monocytogenes* in deli meats exposed to the environment after the lethality treatment has the greatest impact on lowering the risk of illness or death from listeriosis [34].

The Agency used these risk assessments as resources in developing the regulations to control *L. monocytogenes* in RTE meat and poultry products, resulting in the final regulatory document “Control of *Listeria monocytogenes* in Post‐lethality Exposed Ready‐to‐Eat Products (the so‐called *Listeria* Rule).” This *Listeria* Rule codified the regulations that establishments must follow to produce safe RTE products that require no further preparation, like re‐heating. According to the *Listeria* Rule, post‐lethality exposed RTE products are considered adulterated if they contain *L. monocytogenes* or come in direct contact with a food contact surface (FCS) that is contaminated. In other words, there is a zero‐tolerance policy for this type of food product [34].

The *Listeria* rule established three alternative methods to control *L. monocytogenes* contamination of post‐lethality exposed RTE products. The first alternative considers the use of post‐lethality treatment (PLT) to reduce or eliminate *L. monocytogenes*, along with an antimicrobial agent or process (AMAP) to suppress or limit the pathogen growth. The second alternative is the use of either a PLT or an AMAP. The third alternative is not using any PLT or AMAP, instead, relying solely on a sanitation program to control the pathogen. The *Listeria* Rule only applies to RTE products that are exposed to the environment after a lethality treatment (Table 2) [34].

**TABLE 2:** Expected control levels for post‐lethality treatments and antimicrobial agents or processes for establishments that apply a post‐lethality treatment (PLT) to reduce or eliminate *L. monocytogenes* along with an antimicrobial agent or process (AMAP) to suppress or limit its growth, and for establishments that apply either a PLT or an AMAP [34].

Level of control	Increased	Minimum	Not accepted
Post‐lethality treatment (reduction should be achieved prior to distribution of the product into commerce)	2‐logs or greater reduction	At least 1‐log reduction	Less than 1‐log reduction (At this level of reduction, the PLT is not eligible unless there is supporting documentation)
Antimicrobial agent or processes (growth must be limited over the shelf‐life of the product	Allows no more than 1‐log growth	Allows no more than 2‐logs growth	Allows greater than 2‐logs growth (At this level of growth, the AMAP is not eligible unless there is supporting documentation)

For a period of 9 years (2008–2017), the FDA established a limit of 100 CFU/g for RTE that did not support the growth of *L. monocytogenes*. Although the incorporation of *L. monocytogenes* strain virulence variability and host susceptibility into risk assessment studies concluded that low doses of the pathogen may negatively affect susceptible groups if highly virulent strains are present in food. Also, an outbreak of listeriosis in 2015 attributed to ice cream, considered a RTE food that does not support the pathogen growth, and with a low contamination level of *L. monocytogenes* (0.15–7.1 MNP/g), contributed to the agency's decision to reinstate a zero‐tolerance policy for all RTE foods [35, 36, 37].

In the European Union, a zero‐tolerance policy for the presence of *L. monocytogenes* in dairy and ready‐to‐eat products is enforced for foods before they leave the immediate control of the food establishment that has produced them (Commission regulation (EC) N° 2073/2005 amended in 2020). However, levels below 100 CFU/g are allowed for foods other than those intended for infants and for special medical purposes [38, 39].

It was suggested that heat‐treated and preserved foods, when stored under proper conditions, will not support the growth of *L. monocytogenes* during their shelf‐life. For raw ready‐to‐eat foods, a level below 10 CFU/g can be considered acceptable [39]. However, levels between 10 and 100 CFU/g of *L. monocytogenes* are not satisfactory and a level above 100 CFU/g are not acceptable [40, 41]. According to various risk assessments studies, the risk of acquiring listeriosis from RTE products can decrease by 1000 to 10 000‐fold when the growth of the pathogen is prevented [37].

**FIGURE 3:**
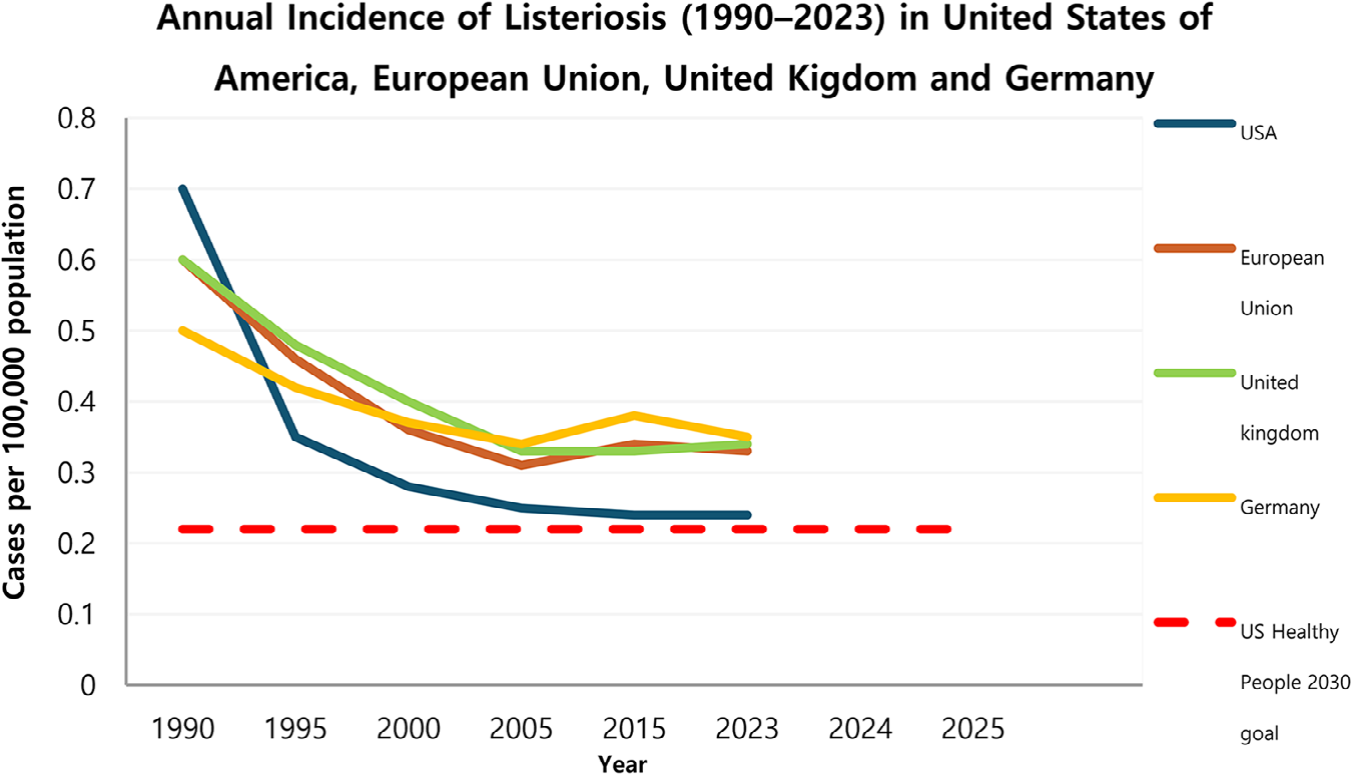
Incidence of reported listeriosis cases in USA (CDC, FoodNET), EU (EFSA, ECDC), UK (UKHSA) and Germany (RKI) from 1993 to 2023. US Healthy People 2030 goal for listeriosis is 0.22 case per 100 000 population (USDHHS).

The question of how many viable cells of *L. monocytogenes* can be present in food is a very polemic topic, since the answer is directly linked to the health status of the consumer and habits to storage conditions of food commodities, and to the presence of other food ingredients that might inhibit or promote pathogen growth. Also, some outbreaks have been associated with foods characterized as not supporting growth of *L. monocytogenes* and that complied with the limit of 100 CFU/g [37, 42]. Despite a more restrictive policy for RTE products, epidemiological data show a stagnation of decreasing incidence rate in the last two decades in United States and countries following de EU policies for *L. monocytogenes* control in RTE products (Figure 3) [43, 44, 45, 46, 47]. This data suggests that *L. monocytogenes* control in food goes beyond more restrictive policies. Besides policies, food industry compliance with Good Manufacturing Practices (GMP), efficient Sanitation Standard Operating Procedure (SSOP), Root Cause Analysis (RCA) to identify the source of environmental and product contamination, along with consumer education are necessary to reduce listeriosis incidence rates [23, 37].

## 3 Conventional and Nonconventional Strategies for Control of *L. monocytogenes*

In addition to conventional technologies for controlling *L. monocytogenes* in the food production chain, such as heat treatment, high‐pressure processing, irradiation, acidification, and drying, alternative strategies are gaining increasing attention. These include the application of specific bacteriophages, use of bacteriocins, photosensitizers and plant extracts, and innovative approaches targeting selective gene expression in L. monocytogenes to regulate bacterial growth and virulence through various modulation strategies. A summary of these alternatives is provided below.

## 4 Biological and Natural Interventions

### 4.1 Bacteriophages

Bacteriophages have emerged as a promising approach in the control of different food‐borne pathogens, gaining attention in both research and industrial applications in the last decades [48, 49, 50]. Bacteriophages are viruses that infect and kill specific bacteria and are divided into two groups according to their characteristics: temperate and lytic phages. The temperate phages are non‐bactericidal and unsuitable for controlling bacterial pathogens as their genetic material can be integrated into the host's cells. In contrast, lytic phages are bactericidal because they replicate within bacterial cells, causing their lysis and release the virion progeny [51, 52].

Discovered over a century ago by William Twort in 1915, and later studied by Felix d'Herelle in 1917, bacteriophages were recognized by their ability to kill bacterial cells [53]. Even being considered as promising tools in treatment of bacterial infection diseases, they were neglected after the discovery and widespread use of antibiotics [54]. This was largely due to their lower effectiveness compared to antibiotics, as well as the time‐consuming preparation and handling required [55].

However, the rise of antibiotic resistance among bacteria has recently revitalized interest in bacteriophages as a potential solution [56, 57]. Bacteriophage research and use continued in the former USSR, where they remain a standard procedure for treatment of various bacterial diseases [58]. Currently the European Union has no specific regulation for the use of phages in human therapy, but since 2019, phages are recognized as a therapeutic option in veterinary medicine [59].

Bacteriophages can be applied to food or food contact surfaces to target and eliminate *L. monocytogenes*. They offer several advantages, including being natural, safe, and host‐specific. Additionally, they do not alter the sensory quality of food products or disrupt the natural food microbiota. However, their application also presents challenges, such as the potential for bacteria to develop phage resistance, the possibility of inducing immune responses in consumers, stability issues in different food matrices, consumers' acceptance concerns and regulatory approval hurdles [48, 49, 50, 52, 56].

In the United States, Australia, Netherlands, Canada, Brazil, Israel, Switzerland, Chile, and New Zealand, the use of phage‐based preparations to control *L. monocytogenes* is allowed for certain RTE products. Despite the recognized efficacy and safety of specific bacteriophage‐based products by the European Food Safety Authority [60], their use has not yet been approved by the EU regulatory agencies. In the absence of a legal framework, the European Court issued a court order in 2019, allowing food companies to continue using a specific bacteriophage‐based product on food contact surfaces to prevent the occurrence of *Listeria* in RTE products [61].

Some studies with commercial bacteriophage‐based products have shown a reduction in *L. monocytogenes* ranging from 0.7 to 8 log CFU/mL, depending on the food matrix. Factors such as pH and temperature can influence the bacteriophages efficacy, and in some cases, their application has inhibited pathogen regrowth [62].

Currently, two commercial bacteriophages‐based products are approved by FDA in USA for control of *L. monocytogenes* in foods: PhageGuard Listex (Micreos, Wageningen, The Netherlands), formerly known as Listex P100, and ListShield (Intralytix, Columbia, USA), a cocktail of phages for RTE foods, surfaces and food environment [63, 64, 65].

Bacteriophages have several advantages as natural control agents for *L. monocytogenes*: (I) They are highly specific and do not affect the beneficial microflora or the sensory properties of food products; (II) They are considered safe for human consumption and do not cause antibiotic resistance or toxic residues; (III) They can be applied at various temperatures, pHs, and salt concentrations, and can penetrate biofilms and niches where *L. monocytogenes* may hide; and (IV) They can multiply and persist in the food environment, providing a long‐lasting protection against *L. monocytogenes* [66].

Bacteriophages have been used to control *L. monocytogenes* in various food products, such as cheese, meat, fish, seafood, fruits, vegetables, and ready‐to‐eat foods [66]. Some examples of bacteriophage commercial products that have been approved or commercialized for this purpose are, ListShield, PhageGuard Listex (formerly Listex P100) [67]. Important point is to be underlain again that bacteriophages are viruses that infect and kill specific bacteria, without harming human, animal, or plant cells [68] and have a great potential to be applied to food or food contact surfaces to target and eliminate *L. monocytogenes* [66].

PhageGuard Listex is a bacteriophage product that contains six different phages that target various strains of *L. monocytogenes*. Most probably PhageGuard Listex can be considered as one of the first bacteriophage products to be Generally Recognized as Safe (GRAS) by the US FDA. It was suggested that PhageGuard Listex can be used to prevent the growth of *L. monocytogenes* in cheese, meat, and vegetable products [68].

ListShield is a bacteriophage product that contains five different phages that target various strains of *L. monocytogenes*. ListShield was approved by the US FDA as a food additive for ready‐to‐eat meat and poultry products. It can be sprayed or dipped onto the surface of the food products to reduce the contamination by *L. monocytogenes* [66].

As principal advantage of bacteriophages, is a fact that those viruses able to infect and kill specific bacteria, are not harming human, animal, or plant cells [69].

Moreover, bacteriophages can affect the microbiota of food products in different ways, depending on their type, target, and application [70], but question if even been characterized as very specific in their antibacterial properties, what about potential negative effects on the commensal GIT microbiota?

Some bacteriophages can be used as natural control agents for foodborne pathogenic and spoilage bacteria, such as *L. monocytogenes*, *Salmonella enterica*, or *Escherichia coli* [69, 70]. These bacteriophages can be applied to food or food contact surfaces to target and eliminate the harmful bacteria, without affecting the beneficial microflora or the sensory properties of food products [69, 70]. This can enhance the safety and quality of food products, including fermented and processed food products such as cheese, meat, fish, seafood, fruits, vegetables, and ready‐to‐eat foods [69, 70].

Some other bacteriophages can be used as microbial source‐tracking and fecal indicators in food products. These bacteriophages can help identify the origin and extent of fecal contamination in food products, such as water, shellfish, or fresh produce. Implementation for such bacteriophages can help assess the risk of exposure to pathogenic bacteria and implement appropriate control measures. However, bacteriophages can also have some challenges and concerns in their use for food products [70]. One of the principal concerns is associated with the fact that bacteriophages may have undesirable effects on the microbiota of food products, such as reducing the fermentation activity of starter cultures or enhancing the virulence of some pathogenic bacteria or simply having negative effect on some beneficial microbes.

Moreover, some bacteriophages may face resistance from the target bacteria or instability in the food environment. Therefore, bacteriophages should be carefully selected and optimized for their specific applications and combined with other strategies to ensure their effectiveness and safety [70]. In 2016, EFSA recommended to undertake experiments to investigate the currently unknown mechanism(s) by which strains of *L. monocytogenes* exhibiting resistance to certain therapeutic antimicrobials become sensitive to these antimicrobials following the development of resistance to Listex TM P100 [60].

Bacteriophages do not affect the sensory properties of food products, such as taste, flavor, color, texture, or aroma [48]. Moreover, it was suggested that bacteriophages can be considered safe for human consumption and do not cause antibiotic resistance or toxic residues [48]. Bacteriophages can affect the shelf life of food products by reducing the microbial load and preventing the growth of spoilage and pathogenic bacteria, including *L. monocytogenes*. This can contribute to and enhance the safety and quality of food products, such as cheese, meat, fish, seafood, fruits, vegetables, and ready‐to‐eat foods [71].

However, bacteriophages may also face some challenges and concerns in their use for food products, such as resistance from the target bacteria or instability in the food environment [71]. Therefore, bacteriophages should be carefully selected and optimized for their specific applications and combined with other strategies, such as acidification, heat treatment, high pressure processing, irradiation, or bacteriocins, to achieve a synergistic effect and ensure the effectiveness and safety of bacteriophage control in food products [71]. It is well reached consensus that bacteriophages do not affect the nutritional value of food products, as they do not alter the chemical composition or the nutritional content of food products [72, 73].

### 4.2 Bacteriocins

Bacteriocins are antimicrobial peptides produced by some bacteria that can inhibit the growth of other bacteria, normally closely related to the producers [74]. Since their discovery in 1925 as antimicrobials produced by *E. coli* [75, 76] and later the identification of nisin, produced by *Lactococcus lactis* [77, 78], these antimicrobial peptides were explored as potential tools for controlling various foodborne pathogens in the food industry [79, 80]. In the last decades, bacteriocins have also gained attention as complementary therapeutical agents for the prevention and treatment of diseases in humans and animals [81].

Some authors suggest that bacteriocins, whether in their natural or postproduction modified forms or developed through gene modification and biotechnological expression, could serve as a promising alternative against antibiotic‐resistant pathogens [80]. Additionally, reports indicate that an expanded range of applications for bacteriocins produced by Gram‐positive bacterial species, with activity against Gram‐negative pathogens and spoilage bacteria, but also against certain viruses, molds, and mycobacteria [74].

Bacteriocins can be used as natural preservatives or bioprotective agents in food to control *L. monocytogenes*, offering the advantage of being effective at low concentrations. However, their use also presents limitations such as a restricted activity range, stability concerns and potential sensory impacts on food products [29, 56, 82]. Their mode of action involves interference with bacterial cell wall synthesis and promotion of pore formation in cell membrane, resulting in permeability changes and loss of essential compounds, such as potassium, amino acids, and ATP, through the pores. This disruption ultimately results in bacterial cell death [83].

Based on their genetic and biochemical characteristics, bacteriocins can be divided into three major classes [84, 85] (Table 3).

**TABLE 3:** Classification of bacteriocins.

Classification	Features	Subcategories	Examples
Class I or lantibiotics	Lantionine or peptides containing β‐lantionine	Type A (linear molecules) Type B (globular molecules)	Nisin, subtilin, epidermine Mersacidin
Class II	Heterogeneous class of small thermostable peptides	Subclass IIa (antilisterial‐pediocine bacteriocins type) Subclass IIb (composed of two peptides) Subclass IIc (other bacteriocins)	Pediocin, enterocin, sakacin Plantaricin, lactacin F Lactococcin
Class III	Large thermolabile peptides	—	Helveticin J, millericin B

*Source*: Drider et al. [84] and Balciunas et al. [85].

Numerous research projects suggest the application of bacteriocins for control of *L. monocytogenes* in dairy, meat, fruit, and vegetables food products [4], and nisin (a bacteriocin produced industrially by *L. lactis*) for control of *Listeria*, other bacterial spoilage and foodborne pathogens [86]. Nisin is produced by some *L. lactis* strains and even by other lactic acid bacteria [87].

Nisin has a broad spectrum of activity against Gram‐positive bacteria, including *L. monocytogenes* and was suggested as appropriate to be applied in preservation of various food products, such as dairy, meat, and canned foods, to prevent the growth of *L. monocytogenes*. Nisin is also approved as a food additive by many regulatory agencies, including EFSA and FDA [88].

The mechanism of action of nisin against *L. monocytogenes* is to dissipate the membrane potential and pH gradient of the bacterial cell. Nisin binds to the cell wall precursor lipid II and forms pores in the cytoplasmic membrane of *L. monocytogenes*, causing the leakage of ions and small molecules. This leads to the loss of electrochemical gradient, energy depletion, and cell death [86, 89, 90].

Specificity of the biochemical properties of nisin, made it very stable at acidic pH and is more heat stable at lower pHs [89]. It can be effective at very low concentrations, such as parts‐per‐billion range [86, 90]. Nisin is produced on an industrial scale and commercialized as Nisaplin [87].

Pediocins are bacteriocins produced by some strains of *Pediococcus* spp. reported to be presenting a narrow spectrum of activity against Gram‐positive bacteria, especially *L. monocytogenes* that can be applied to food products, including meat, cheese, and fermented vegetables, to inhibit the growth of *L. monocytogenes*. Pediocin is also considered as a generally recognized as safe (GRAS) substance by the FDA [7, 91]. Moreover, pediocin PA‐1 was produced at industrial scale and commercialized as Alta 2341 [92].

Even if only nisin and pediocin PA‐1 are commercially produced, authorized in several countries as safe additives for control of foodborne and spoilage pathogens [92], other bacteriocins are in the pipeline for the commercialization, based on solid scientific evidence for their effectiveness in control of pathogens and safety for the consumers.

Listeriolysin S is a bacteriocin produced by some strains of *L. monocytogenes* itself and that presents a specific activity against other strains of *L. monocytogenes* and can help the producer strain to outcompete its rivals in the same niche. Lamentably, listeriolysin S can also affect the composition of the intestinal microbiota and enhance the virulence of *L. monocytogenes*. Therefore, listeriolysin S is considered by some authors as a novel virulence factor of *L. monocytogenes* [56] and more research is needed before being applied as a safe additive in food processing practices.

There are some enterocins, plantaricins, and lactocins that have been applied in the control of *L. monocytogenes* in food products within numerous research projects. These are bacteriocins produced by different strains of lactic acid bacteria that can inhibit the growth of *L. monocytogenes* and other pathogens [93]. Some examples are the enterocins, produced by numerous *Enterococcus* spp. However, as Chikindas et al. [94] suggested, it is possible that bacteriocins can be involved in more physiological bacterial processes, than just simple killing other microbial species. The role of bacteriocins as *quorum sensing* signaling molecules, involved in signaling processes, was suggested in recent years [94].

Enterocin A is a bacteriocin produced by *Enterococcus faecium* strains. According to scientific reports, this bacteriocin has a broad spectrum of activity generally against Gram‐positive bacteria, including *L. monocytogenes*. It was suggested that enterocin A can be used to prevent the growth of *L. monocytogenes* in cheese, meat and vegetable products [93, 95].

Enterocin AS‐48 is one of the few bacteriocins with cyclic polypeptide molecules [96]. This is a bacteriocin produced by *E. faecalis* strains presenting a broad spectrum of activity against Gram‐positive including *L. monocytogenes* and even some Gram‐negative bacteria. Enterocin AS‐48 can be used to prevent the growth of *L. monocytogenes* in cheese, meat, and fruit products, as reported in different research studies [93, 97].

Plantaricins are bacteriocins produced by *Lactiplantibacillus* (former *Lactobacillus*) *plantarum* strains. Examples of some specific plantaricins are plantaricin A, a bacteriocin with a narrow spectrum of activity against *L. monocytogenes* and other closely related bacteria. Plantaricin A was suggested as an effective bacteriocin for prevention of the growth of *L. monocytogenes* in cheese and meat products [93]. Another example, plantaricin E, was reported to present a broad spectrum of activity against Gram‐positive, including *L. monocytogenes*, and even some Gram‐negative bacteria. Plantaricin E was suggested to be applied in the prevention of the growth of *L. monocytogenes* in cheese and vegetable products [93, 95].

Lactocins S is a bacteriocin produced by some *Latilactobacillus* (former *Lactobacillus*) *sakei* strains. This bacteriocin was reported to present a narrow spectrum of activity against *L. monocytogenes* and other closely related bacteria. Some reports suggest that lactocin S can be used to prevent the growth of *L. monocytogenes* in meat and fish products [93, 98].

Lactocin AL705 is a *quorum sensing* bacteriocin produced by *Lacticaseibacillus* (former *Lactobacillus*) *casei* strains. Lactocin AL705 was reported to present a broad spectrum of activity against Gram‐positive, including *L. monocytogenes* and some Gram‐negative bacteria. It was suggested that lactocin AL705 can be used to prevent the growth of *L. monocytogenes* in dairy products [93, 99].

Different strains of *Lactococcus* can be mentioned as bacteriocin producers. Most iconic example is nisin, belonging to the lantibiotics group of bacteriocins. Specificity of lantibiotics is due to the presence of some modified amino acids in their polypeptide structure [86]. Different from lantibiotics, without involve modified amino‐acid in the structure of antimicrobial, are some other lactococcal bacteriocins, including those produced by *Lactococcus garviae* strains [100], with application for inhibition of *L. monocytogenes* [101].

Mundticin and carnocin are two examples of bacteriocins that have been applied in the control of *L. monocytogenes* in food products [56]. Mundticin is a bacteriocin produced by *Enterococcus mundtii* strains. Mundticin has a broad spectrum of activity against Gram‐positive bacteria, including *L. monocytogenes*. Mundticin can be used to prevent the growth of *L. monocytogenes* in cheese, meat, and vegetable products [66, 102, 103].

Carnocins are a bacteriocin produced by *Carnobacterium piscicola* strains. They were described as narrow spectrum bacteriocins with activity against *L. monocytogenes* and other closely related bacteria. Carnocins were suggested as potential preservatives to be used to prevent the growth of *L. monocytogenes* in fish and seafood products [56, 97].

Both, mundticins and carnocins, have potential for application as natural preservatives or bioprotective agents in foods to control *L. monocytogenes* and enhance the safety and quality of food products. However, they may also have limitations such as stability, activity range, or sensory impact. Therefore, they should be combined with other strategies, such as acidification, heat treatment, high pressure processing, irradiation, or bacteriophages, to achieve a synergistic effect and ensure the effectiveness of *L. monocytogenes* control in food products [56, 66, 97, 102].

### 4.3 Antibiotics—An Alternative That Was, But Not Anymore an Option

Although antibiotics are well known for their antimicrobial properties, including their effectiveness against *Listeria*, they are not a recommended option for the control of *L. monocytogenes* in the food industry. Their use in farming practices and food production is banned in several countries due to concerns over antibiotic resistance development in bacterial populations. Additionally, antibiotics can negatively impact food quality and safety, altering flavor, texture, or color, or they may pose health risks such as allergic reactions or contributing to antibiotic resistance in consumers [104].

Moreover, antibiotics may not be effective in fully eliminating *L. monocytogenes* from food or food processing environments, as this bacterium can develop antibiotic resistance or form biofilms that protect it from antibiotic exposure [105]. Therefore, the food industry should prioritize alternative control methods. As previously discussed, good hygiene practices, listericidal steps, bacteriophages, and bacteriocins offer more natural, safe, and targeted approaches. These methods can significantly minimize or prevent contamination of ready‐to‐eat foods with *L. monocytogenes* while reducing the risks associated with antibiotic use [106].

### 4.4 Plant Extracts

Certain spices, commonly added to foods to enhance the flavor and quality, can be an option for control of *L. monocytogenes* in specific food products due to their antimicrobial properties [66, 107]. Some examples are garlic, onion, cinnamon, clove, oregano, thyme, rosemary, and sage [66, 107].

These spices contain various bioactive compounds, such as allicin, eugenol, carvacrol, thymol, and rosmarinus acid, that can disrupt bacterial cell membrane, interfere with enzyme activity, or even affect gene expression of *L. monocytogenes* [60, 108]. However, spices alone may not be sufficient to control *L. monocytogenes* in food products, as they may have limited effectiveness or undesirable sensory effects when used in high concentrations [60].

To achieve more effective control, spices should be combined with other methods, such as acidification, heat treatment, high pressure processing, or irradiation. This combination can achieve a synergistic effect, enhancing both the safety and quality of food products [4, 60].

Curcumin, a natural compound derived from turmeric, possesses antimicrobial properties and has been shown to inhibit the growth of *L. monocytogenes* in food products [109]. Curcumin targets the pore‐forming toxin listeriolysin O (LLO), which is an essential virulence factor of *L. monocytogenes*, by reducing its oligomerization and hemolytic activity [109]. Additionally, curcumin has been suggested to enhance the clearance of *L. monocytogenes* by macrophages, offering protection against infection in mice [109].

Curcumin can be added to food products as a natural preservative or bioprotective agent to control *L. monocytogenes*. For example, it can be incorporated into edible films or coatings to prevent the post‐processing contamination of ready‐to‐eat foods [110]. Moreover, curcumin can also be encapsulated into nanoparticles or liposomes to improve its stability and delivery in food systems [111, 112].

However, as with other natural antimicrobials, curcumin alone may not be sufficient to control *L. monocytogenes* in food products, as it may have limited effectiveness or undesirable sensory effects at high concentrations. Therefore, curcumin should be combined with other strategies, such as acidification, heat treatment, high pressure processing, irradiation, or bacteriophages, to achieve a synergistic effect and ensure the safety and quality of food products [97, 109, 111, 112].

### 4.5 Conventional Nonbiological Control Strategies

### 4.6 Heat Treatment

Heat treatment applied in the food industry is a process that can reduce the presence of different microbial contaminants, including *L. monocytogenes*. From one side, this is a well applied approach in the food industry. However, in some cases, high temperature treatments can have negative effects by reducing beneficial properties of foods such as vitamins and bioactive peptides, changing sensory quality, producing Maillard reactions and formation of some potentially harmful Maillard reactions products (advanced glycation end products) and, even promote spread of some bacterial groups [113]. Alternatives to heat treatments have always been a subject for exploring within the food industry, searching for more natural and minimal processed food products [114]. However, heat treatment is one of the most applied approaches for the control of microbial contaminants in the food industry. Heat treatment can be applied to raw materials or finished products, depending on the type of food and the level of contamination.

The effectiveness of heat treatments depends on several factors, such as the temperature, time, pH, water activity, growth phase, exposure to sublethal stress and presence of other microorganisms or substances that may protect or inhibit *L. monocytogenes* [115, 116].

Basically, the heat treatment affects cellular structures such as the outer and inner membrane, the peptidoglycan cell wall, the nucleoid, the cell's RNA, ribosomes, and enzymes resulting in bacterial inactivation. Dry heat oxidizes the bacterial structures while moist heat promotes denaturation and oxidative stress, and is the main method used in the food industry. Both methods disrupt bacterial homeostasis and basic functions leading to bacterial inactivation. The damage caused by the heat treatment to the structures must be at a certain level that prevents the bacterial repair. Gram‐positive bacteria are more resistant to heat due to their thicker cell wall compared to Gram‐negative but with some exceptions [117, 118].

One way to measure the effectiveness of heat treatment is by using the concept of lethal rate, which is the relative lethality of 1 min at a given temperature compared to 1 min at a reference temperature. For example, the lethal rate for *L. monocytogenes* at 60°C is 10, which means that 10 min at 60°C is equivalent to 1 min at the reference temperature of 121.1°C [119]. F0 value is the time in minute for the specified temperature that gives the same thermal lethality as at 121.1°C in 1 min.

Another way to measure the effectiveness of heat treatment is by using the concept of decimal reduction time (*D*‐value), which is the time required to reduce the population of *L. monocytogenes* by 90% (or one log cycle) at a given temperature. For example, the *D*‐value for *L. monocytogenes* in milk at 72°C is 0.5 min, which means that it takes 0.5 min to reduce the population of *L. monocytogenes* by 90% at 72°C [119].

Heat treatment can be an effective method to control *L. monocytogenes* in food production processes, but it should be combined with other strategies to prevent cross‐contamination or recontamination after treatment. Heat‐treated foods should be stored and handled properly, and the food processing environment should be cleaned and sanitized regularly [12].

### 4.7 High Pressure Processing (HPP)

High pressure treatment, also known as HPP, is a non‐thermal technique that uses hydrostatic pressure to inactivate pathogens and spoilage microbes in food products. HPP can be applied to various types of food, such as meat, dairy, fruit and vegetable products, without affecting their nutritional or sensory qualities when compared to conventional thermal treatments. HPP can also extend the shelf life of food products via reducing microbial load and enzymatic activity [120, 121].

The treatment consists of application of isostatic pressure (equal pressure in all points within the vessel) using a liquid (water, glycol‐water solution, and propylene with glycol) to transmit the pressure to the food in a vessel. The treatment is usually applied with food packages, avoiding post‐treatment contamination. Usually uses pressures ranging from 400 to 600 MPa, a holding time of 1–15 min, and temperatures varying from 10°C to 40°C. Gram‐positive microorganisms and spores are more resistant to HPP when compared to Gram‐negative, yeasts, and molds [122, 123].

Microbial inactivation by HPP is due to a sum of deleterious effects on microbial cell membrane, cell wall, biochemical reactions, and genetic mechanisms. The cell membrane is the most structure affected by HPP, resulting in membrane permeability and outflow of important compounds, for example, Adenosine Triphosphate (ATP), and osmolarity imbalance.

Denaturation of proteins and enzymes caused by HPP interferes, not only in cell membrane and wall, but also in different biochemical reactions, disrupting ATP production, replication and transcription of DNA, and many others functions that are enzyme dependent, leading to microbial inactivation [122, 123].

The implementation and effectiveness of HPP depends on several factors, such as pressure level, treatment time, temperature, pH, water activity and the type of food matrix [120, 121, 124].

Generally, higher pressure levels and longer treatment times result in greater microbial inactivation. However, as previously mentioned, some microorganisms may be more resistant to HPP than others. For example, *L. monocytogenes* is one of the most pressure‐resistant bacteria [120, 121].

Therefore, if aimed at controlling *L. monocytogenes* in food products by HPP, it is relevant to optimize the processing parameters and even combine HPP with other strategies, including adding antimicrobial agents or applying other preservation methods [120, 121, 125].

Some of the appropriate examples to join HPP applications are antimicrobial agents (such as organic acids, bacteriocins, bacteriophages, or reuterin) that can enhance the effect of HPP [120, 125, 126]. Moreover, other preservation methods that can be applied in combination with HPP and improve preservation effectiveness are heat treatment, irradiation, ultraviolet light, and photocatalysis [120, 125, 126].

### 4.8 Irradiation

Irradiation is a non‐thermal technique that uses ionizing and non‐ionizing radiation to inactivate pathogens and spoilage microbes in food products [30, 56]. Irradiation can be applied to various types of food, such as meat, poultry, seafood, fruits and vegetables, without affecting their nutritional or sensory properties. Irradiation can also extend the shelf‐life of food products by reducing the microbial load, germination and sprouting of vegetables, and food enzymatic activity [30, 56].

Ultraviolet radiation, infrared, microwaves and radio frequency are some examples of non‐ionizing radiation and are characterized by low energy and long wavelengths (>100 nm). Ultraviolet radiation is the most used, and can be applied to foods, surfaces and utensils for microbial decontamination. The non‐ionizing radiation inactivates microorganisms by damaging the cell membrane, DNA and RNA. Ionizing radiation has short wavelengths and higher energy that is responsible for removing an electron from an atom and forming an ion, causing direct and indirect effects on microbial cells. The direct effect is by damaging carbohydrates, DNA, RNA and lipids; the indirect effect is caused by free radicals and reactive oxygen species (ROS) produced during water radiolysis that will react with different cellular components [127].

However, the effectiveness of irradiation for food preservation depends on several factors, including the source, the temperature, the pH, the water activity and the type of food matrix [30, 56]. Generally, combination of higher doses of irradiation and lower temperatures result in greater microbial inactivation, and the food industry actively apply this approach. However, some microorganisms may be more resistant to irradiation than others. For example, *L. monocytogenes* is one of the most radiation‐resistant bacteria within food borne pathogens and spoilage microbes [30, 56].

Therefore, to effectively control *L. monocytogenes* in food products by application of irradiation, it is important to optimize the processing parameters and, in addition to irradiation, apply supplementary preservation strategies, such as adding antimicrobial agents or applying other preservation methods [30, 56].

Some examples of antimicrobial agents that can enhance the effect of irradiation are organic acids, bacteriocins, bacteriophages, and some spices with antimicrobial properties [56]. Moreover, some other examples of other preservation approaches that can have synergetic interactions with irradiation are heat treatment, high pressure processing, ultraviolet light, and modified atmosphere packaging [56].

### 4.9 Acidification and pH Control

One of the most traditional and widely applied concepts in foods preservation for controlling microbial spoilage and pathogens is lowering pH levels [128]. In traditional fermentation processes, conducted by lactic acid bacteria, preservation is associated with drop of the pH and presence of lactic and other organic acids [115], a process, based on empiric knowledge, used for centuries [71]. In industrial food processes, acidification is a technique that uses organic acids or acid‐producing microorganisms to lower the pH of food products and inhibit the growth of pathogens and spoilage microbes [56, 97].

Acidification can be applied to various types of food, such as dairy, meat, fruit and vegetable products, to enhance their flavor, texture, and actively contributing to their safety by reducing (or even eliminating) some of the food spoilage and pathogenic bacterial species. Acidification can also extend the shelf life of food products by reducing the water activity and enzymatic activity [56, 97].

However, as has been stated for most of the previously discussed factors involved in the preservation processes, the effectiveness of acidification depends on several factors, such as the type, concentration, and distribution of the acid, the pH, the temperature, the water activity and the type of food matrix [56, 94].

Generally, lower pH and higher acid concentration result in greater microbial inactivation. However, some microorganisms may be more resistant to acid than others. For example, *L. monocytogenes* is one of the most acid‐tolerant bacteria [30, 56, 97]. Therefore, to control *L. monocytogenes* in food products by acidification, it is important to optimize the processing parameters and combine acidification with other strategies, such as adding antimicrobial agents or applying other preservation methods [4, 56, 97]. Some examples of antimicrobial agents that can enhance the effect of acidification are salt, nitrite, lactate, diacetate, or bacteriocins [30, 97]. Some examples of other preservation methods that can synergize with acidification are heat treatment, high pressure processing, irradiation, or modified atmosphere packaging [56, 97].

### 4.10 Water Activity

Water activity (*a_w_*) is a measure of the availability of water for microbial growth in food products. Lowering the *a_w_* can contribute to the control of *L. monocytogenes* by limiting its growth and survival in food products [12, 30, 129]. According to FDA guidance document [130], the minimum *a_w_* for *L. monocytogenes* growth is 0.90, which means that food products below 0.90 can prevent the growth of this pathogen. The problem is that *L. monocytogenes* can still survive for extended periods of time, even at *a_w_* values as low as 0.81, as shown by Nolan et al. [131] and later, when environmental conditions are favorable, the pathogen can recover.

Therefore, a_w_ alone may not be sufficient to control *L. monocytogenes* in food products, and other factors such as pH, salt, temperature, and antimicrobial agents should also be applied in combination [30, 129].

Some examples of food products that have low a_w_ values, presenting low risk of presence of *L. monocytogenes* are dried fruits, nuts, honey, jams, jellies, and some hard cheeses [12, 129]. However, these products may still be contaminated with *L. monocytogenes* from the environment or other sources and may pose a risk to consumers if they are rehydrated or consumed by susceptible people. Therefore, good hygiene practices and proper storage and handling are also important to control *L. monocytogenes* in food products with low *a_w_* values [12, 129].

## 5 Emerging Technologies

### 5.1 Photosensitizers

Photosensitizers are metabolites or substances that can absorb light and, consequently, initiate a photochemical reaction. They trigger intracellular processes by either donating electrons to a substrate or abstracting a hydrogen atom from it. After completing this process, photosensitizers return to their ground state, ready for the next cycle and absorb more light. From a practical point of view, they have applications in photodynamic therapy, photocatalysis, and photon up conversion [132].

Additionally, photosensitizers have been proposed as an effective tool for the control of *L. monocytogenes* through a process known as photodynamic inactivation (PDI). This method involves the use of photosensitizing compounds that, when exposed to specific wavelengths of light, generate ROS. These ROS, in turn, induce damage to bacterial cellular components, disrupting vital functions and ultimately leading to bacterial inactivation and death [132].The advantages of PDI in controlling *L. monocytogenes* are that (i) photosensitizers can be selected or designed with high specificity to target selected bacterial structures, ensuring that the ROS generated will effectively disrupt the bacterial vital functions; (ii) unlike traditional antibiotics, PDI is less likely to promote resistance, as the ROS produced can damage multiple bacterial targets simultaneously; and (iii) PDI can be successfully applied to various food processing environments, including factory surfaces where *L. monocytogenes* may be present, making it a versatile tool for controlling contamination [133].

The use of photosensitizers in the food industry to control *L. monocytogenes* and other foodborne pathogens is an active research area. This research explores the potential of different photosensitizing compounds and light sources, aiming to optimize the inactivation process and being a winner step of combating pathogens. While it is seen as a promising approach in food processing and preservation, it is still considered more effective when combined with other existing hygiene and safety practices for the reduction and/or elimination of *L. monocytogenes* and other foodborne pathogens [30, 102].

The food industry can effectively apply PDI to control *L. monocytogenes* by integrating it into existing food preservation processes. This can be achieved by selecting natural photosensitizers that are active against *L. monocytogenes* and safe for use in food products, choosing light sources that activate the selected photosensitizers without compromising the food product or beneficial microbes and optimizing the PDI parameters, such as light intensity and exposure time, to maximize inactivation while preserving food quality. Additionally, PDI can be explored for various food matrixes, including fresh vegetables, fruits, seafood, and poultry, to reduce microbial load. It is crucial to ensure that the PDI process does not compromise the nutritional and sensory qualities of the food, does not interfere with beneficial microbial cultures, while still adhering to safety regulations [133].

PDI can be a non‐toxic, low‐resistance method to enhance food safety and extend shelf life, aligning with consumer demand for natural and safe food preservation methods [134, 135]. One promising example is riboflavin‐5′‐phosphate (R‐5‐P), which when combined with a specific light wavelength, can significantly reduce bacterial biofilms on food contact surfaces [134].

A review by Zhu et al. [135] highlights the rapid adoption of PDI technology in food safety, emphasizing the recent advancement in developing PDI treatments for various foodborne microorganisms, discussing the mechanisms, influencing factors, and application of different photosensitizers in various food substrates.

### 5.2 Control of Gene Expression

An alternative approach to controlling *L. monocytogenes* growth and virulence involves selective gene expression, which can be achieved through various strategies. External signals are crucial for bacterial behavior, allowing bacteria to sense their environment and adjust gene expression accordingly. Over time, evolutionary processes have selected mechanisms that enhance adaptation and survival, even for pathogens. For instance, the presence of chitin has been shown to actively downregulate the expression for virulence genes in *L. monocytogenes* [136, 137].

Additionally, metabolites known as nucleomodulins are bacterial‐produced factors that can manipulate host cell biology, including altering chromatin structure, which in turn affects gene expression. This manipulation can influence the infection process and the bacteria's ability to cause disease [5].

Control of RNA and Protein‐Mediated interactions play a key role in regulating virulence genes and can be achieved through RNA molecules and proteins that govern gene expression. For example, the motility gene repressor MogR and the glycosyltransferase and motility anti‐repressor GmaR antagonize each other and, consequently, they are able to control the production of flagellin, a critical component for bacterial motility [136]. Moreover, applying selective pressures, such as targeted antimicrobials or bacteriophages, can induce changes in gene expression, as bacteria attempt to adapt to these new threats.

Specific genetic engineering techniques such as CRISPR‐Cas9 can be used to edit the genome of *L. monocytogenes*, potentially knocking out genes essential for its virulence or survival, allowing a deep understanding of the factors that can influence targeted gene expression in *L. monocytogenes*, given possibility to develop targeted approaches to control this pathogen and reduce the risk of foodborne illnesses. These strategies can be particularly useful in the food industry, where controlling this pathogenies is crucial for ensuring food safety [5, 136, 137].

Effective strategies to influence gene expression aiming to control *L. monocytogenes* in food processing involve a multi‐faceted approach, including adjusting environmental factors (temperature, pH, and/or salinity) to influence gene expression in *L. monocytogenes*, thereby affecting its ability to survive and grow [138].

Biofilm management has always been a particular focus in the food industry, as *L. monocytogenes* can form biofilms that protect them from environmental stresses. Implementing cleaning and sanitation protocols that disrupt biofilm formation is a key goal [12].

Bacteriophages (natural or modified) can be applied to target specific strains of *L. monocytogenes*, altering their gene expression, and reducing their virulence or survivability [12]. Alternatively, the application of competitive microorganisms, such as specific strains of lactic acid bacteria that can effectively suppress the growth of *L. monocytogenes* through competitive exclusion and production of antimicrobial compounds, are just some of the possible means for biological control of the pathogen [74, 86].

New approaches based on technologies that create hurdles for bacterial survival, such as high‐pressure processing or pulsed electric fields, can stress the bacteria and affect gene expression related to survival and/or virulence. Regular monitoring of *L. monocytogenes* levels and gene expression profiles can help in early detection and implementation of control measures and should be consistently applied [12].

There are several successful case studies where biomolecular manipulation and gene expression control have been applied in food processing. It was suggested that CRISPR/Cas in Crop Improvement can be a way of controlling *L. monocytogenes* where the application of CRISPR/Cas genome editing technology can be implemented in the processes of regulation of gene expression in crops. This technology has been used to enhance crop genetic diversity and improve traits such as yield, quality, and resistance to environmental stresses [139, 140]. Moreover, genetic control strategies for bioprocesses involving practical genetic control strategies have been developed for industrial bioprocesses, which are crucial to producing food ingredients and additives [140].

From regulations perspective genetically modifications of microorganisms and their applications in food production and safety is not permitted, however, academic research projects can be interesting approaches for exploring these possibilities. With this perspective, CRISPR/Cas genome editing technology can be one of the potentials tools where by modifications in beneficial microbes can be reaching better efficacity in the inhibition/killing spoilage and pathogens or creating competitive “pathogens” with no virulence or spoilage potential. Moreover, research tool potentials of CRISPR/Cas can be appropriate approach for evaluating virulence mechanisms or as a detection/diagnostic strictly academic models studies, rather than as a direct control strategy with industrial applications.

## 6 Conclusions

The control of *Listeria monocytogenes* in RTE products remains a persistent challenge due to its ubiquitous environmental distribution, adaptive physiology and high virulence of certain strains. Its control goes beyond more restrictive regulatory policies. Effective control depends on the food industry's full compliance with GMP, SSOP, effective monitoring program, and RCA. In addition, consumer education initiatives play a key role in mitigating risks at the point of consumption. Transcriptomics approaches are also proving valuable for the discovery and development of novel antimicrobials that interfere with gene expression, especially virulence genes. Continued research into innovative technologies and the integration of multiple control strategies remains essential—particularly those aimed at preventing post‐lethality contamination in RTE products.
